# Proteomic profiling of small-molecule inhibitors reveals dispensability of MTH1 for cancer cell survival

**DOI:** 10.1038/srep26521

**Published:** 2016-05-23

**Authors:** Tatsuro Kawamura, Makoto Kawatani, Makoto Muroi, Yasumitsu Kondoh, Yushi Futamura, Harumi Aono, Miho Tanaka, Kaori Honda, Hiroyuki Osada

**Affiliations:** 1 Chemical Biology Research Group, RIKEN Center for Sustainable Resource Science (CSRS), 2-1 Hirosawa, Wako, Saitama 351-0198, Japan

## Abstract

Since recent publications suggested that the survival of cancer cells depends on MTH1 to avoid incorporation of oxidized nucleotides into the cellular DNA, MTH1 has attracted attention as a potential cancer therapeutic target. In this study, we identified new purine-based MTH1 inhibitors by chemical array screening. However, although the MTH1 inhibitors identified in this study targeted cellular MTH1, they exhibited only weak cytotoxicity against cancer cells compared to recently reported first-in-class inhibitors. We performed proteomic profiling to investigate the modes of action by which chemically distinct MTH1 inhibitors induce cancer cell death, and found mechanistic differences among the first-in-class MTH1 inhibitors. In particular, we identified tubulin as the primary target of TH287 and TH588 responsible for the antitumor effects despite the nanomolar MTH1-inhibitory activity *in vitro*. Furthermore, overexpression of MTH1 did not rescue cells from MTH1 inhibitor–induced cell death, and siRNA-mediated knockdown of MTH1 did not suppress cancer cell growth. Taken together, we conclude that the cytotoxicity of MTH1 inhibitors is attributable to off-target effects and that MTH1 is not essential for cancer cell survival.

Elevated levels of reactive oxygen species (ROS) are a common characteristic of many types of cancers^1^. While altered redox regulation may promote cancerous phenotypes such as excessive proliferation and angiogenesis^2^,^3^, ROS can adversely affect cancer cells by indiscriminately damaging biomolecules, including both DNA and free bases in the nuclear and mitochondrial pool of deoxynucleoside triphosphate (dNTP)^4^. The free dNTP precursor pool is considerably more sensitive to damage compared to that of double-stranded DNA^5^, but incorporating oxidized nucleotides into DNA can cause DNA mutations and cell death. Therefore, preventing accumulation and incorporation of oxidized nucleotides in DNA is crucial for cancer cell survival.

The protein MTH1 (also known as NUDT1) is a Nudix family hydrolase that converts oxidized purine nucleoside triphosphates, including 8-oxo-2′-deoxyguanosine-5′-triphosphate (8-oxo-dGTP) and 2-hydroxydeoxyadenosine 5ʹ-triphosphate (2-OH-dATP), into the corresponding nucleoside monophosphates, thereby preventing utilization of these oxidized nucleotides by DNA polymerases and incorporation into DNA^6^. Rai *et al.* reported that overexpression of MTH1 protected primary fibroblasts from *RAS*-induced senescence by suppressing DNA damage caused by ROS^7^. In addition, it was reported that cancer cells frequently overexpress MTH1, and that inhibition of MTH1 by genetic and pharmacologic methods suppressed xenograft tumors derived from a number of different tumor cell lines^8^,^9^,^10^. Four types of first-in-class small-molecule inhibitors of MTH1 were recently reported—TH287/TH588, (*S*)-crizotinib, SCH51344, and organometallic complexes—among which TH287/TH588, (*S*)-crizotinib, and SCH51344 preferentially kill cancer cells or *RAS*-transformed fibroblast cells^8^,^9^,^11^. Based on these reports, MTH1 has attracted attention as a potential molecular target for the treatment of cancers with elevated ROS levels.

We started the screening of MTH1 inhibitors using chemical array platforms, ultrahigh throughput screening for small-molecule binders of proteins of interest^12^,^13^,^14^. Our chemical arrays were used to obtain small-molecule inhibitors of various proteins derived from mammals^15^,^16^,^17^,^18^,^19^,^20^,^21^, fungi^22^, prokaryotes^23^, and viruses^24^,^25^.

We previously established and utilized ChemProteoBase profiling method to predict the modes of action of bioactive compounds^26^,^27^,^28^,^29^,^30^,^31^. This method allows us to unravel the hidden target of the inhibitors reported as MTH1 inhibitors.

## Results

### Identification of new MTH1 inhibitors

**Figure 1:**
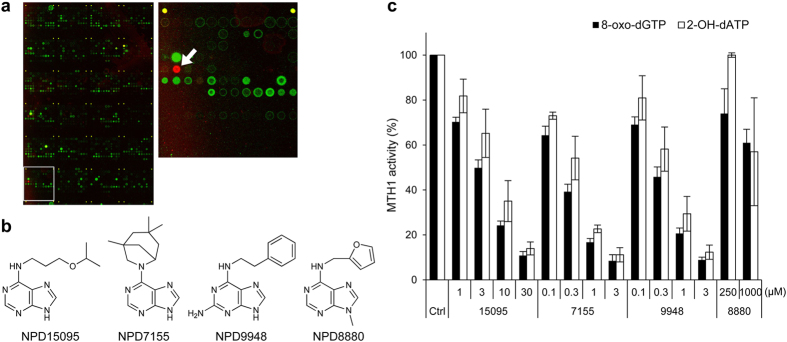
Identification of purine-based MTH1 inhibitors by chemical array screening. (**a**) Representative fluorescent image of the chemical arrays (left) and magnified image of the area in a white square (right). Spot of NPD15095 is indicated by a white arrow. (**b**) Chemical structures of purine-based MTH1 inhibitors. (**c**) Effects of NPD15095 (15095), NPD7155 (7155), NPD9948 (9948), and NPD8880 (8880) on the catalytic activity of MTH1. 8-oxo-dGTP and 2-OH-dATP were used as substrates. Data are shown as mean ± s.d. from three independent experiments.

To identify new MTH1 inhibitors, we used chemical arrays to screen 29,809 compounds from a chemical library of the RIKEN Natural Products Depository (NPDepo)^32^,^33^. Twenty MTH1 binders were detected (Fig. 1a). Next, we ran an *in vitro* enzyme assay to determine the inhibitory effects of these screening hits against MTH1, with 8-oxo-dGTP and 2-OH-dATP as substrates. Subsequently, we found that NPD15095, a purine derivative, inhibited MTH1 catalytic activity in a dose-dependent manner (Fig. 1b,c). The IC_50_ values of NPD15095 for MTH1 inhibition were 3.3 μM and 6.7 μM, for 8-oxo-dGTP and 2-OH-dATP, respectively.

To find more potent MTH1 inhibitors, we explored 131 structurally related compounds with a purine moiety. Two potent MTH1 inhibitors (NPD7155 and NPD9948) and a less active analog (NPD8880) were discovered (Fig. 1b,c). The potencies of NPD7155 and NPD9948 were comparable to those of (*S*)-crizotinib and SCH51344 (Supplementary Table S1). Therefore, we focused on NPD7155 and NPD9948 for further validation studies.

**Figure 2:**
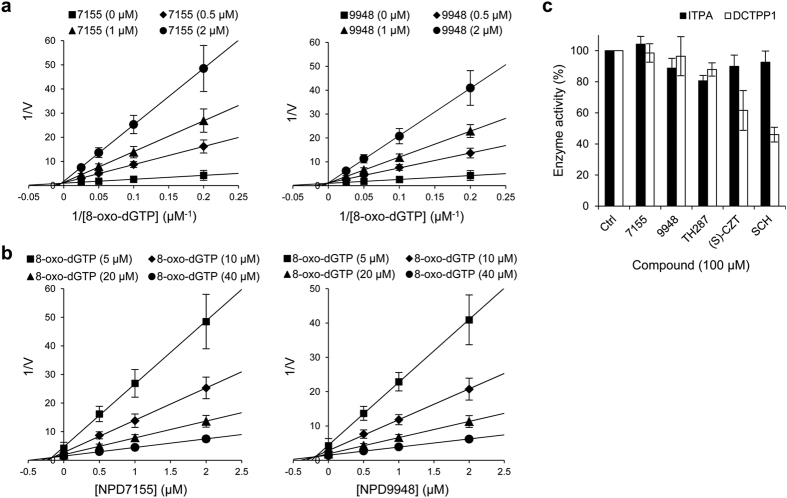
Kinetics and selectivity of NPD7155 and NPD9948. (**a**,**b**) Kinetic analysis of NPD7155 (7155) and NPD9948 (9948) against MTH1. Lineweaver–Burk plot of reciprocal of initial velocity vs. reciprocal of varying 8-oxo-dGTP concentrations (**a**) and Dixon plot of reciprocal of initial velocity vs. varying concentrations of NPD7155 or NPD9948 (**b**). Data are shown as mean ± s.e.m. from three independent experiments. (**c**) Effects of NPD7155, NPD9948, TH287, (*S*)-crizotinib [(S)-CZT], and SCH51344 (SCH) on the catalytic activities of ITPA and DCTPP1 *in vitro*. Data are shown as mean ± s.d. from three independent experiments.

Kinetic analysis showed that both NPD7155 and NPD9948 inhibited the enzymatic activity of MTH1 in a competitive manner with respect to 8-oxo-dGTP and 2-OH-dATP (Fig. 2a and Supplementary Fig. S1a). The *K*_i_ values of NPD7155 and NPD9948 were 0.10 ± 0.04 μM and 0.13 ± 0.05 μM for 8-oxo-dGTP, and 0.18 ± 0.03 μM and 0.22 ± 0.04 μM for 2-OH-dATP, respectively (Fig. 2b and Supplementary Fig. S1b).

As NPD7155 and NPD9948 are mimics of the purine moiety of MTH1 substrates such as 8-oxo-dGTP and 2-OH-dATP, we examined their effects on other nucleoside triphosphate pyrophosphatases, such as inosine triphosphate pyrophosphatase (ITPA) and deoxycytidine triphosphate pyrophosphatase 1 (DCTPP1), whose substrates are purine nucleotides and pyrimidine nucleotides, respectively. In our results, NPD7155 and NPD9948 did not affect the enzymatic activities of ITPA and DCTPP1, even at 100 μM. In contrast, (*S*)-crizotinib and SCH51344 slightly inhibited DCTPP1 activity at the same concentration, suggesting NPD7155 and NPD9948 have some more target selectivity than the inhibitors (Fig. 2c).

### Purine-based MTH1 inhibitors exhibit less potent cytotoxicity

**Figure 3:**
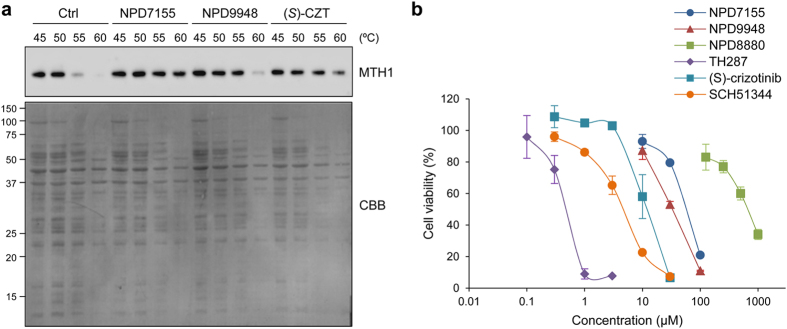
NPD7155 and NPD9948 exhibit less potent cytotoxicity. (**a**) Target engagement of NPD7155 and NPD9948 to MTH1 protein in intact HeLa cells. HeLa cells were treated with NPD7155 (300 μM), NPD9948 (300 μM), or (*S*)-crizotinib (30 μM) for 1 h, collected, heated at the indicated temperatures, and lysed. Soluble proteins collected in the supernatant were subjected to western blot analysis to detect MTH1, followed by staining with Coomassie brilliant blue (CBB). (**b**) Viability of HeLa cells treated with MTH1 inhibitors for 72 h. Data are shown as mean ± s.d.

We next examined the effects of NPD7155 and NPD9948 on MTH1 in cancer cells. To determine whether NPD7155 and NPD9948 target MTH1 in intact cells, we performed a target engagement assay based on ligand-increased thermal stabilization^34^. Treatment of human cervical cancer HeLa cells with NPD7155 and NPD9948 considerably stabilized cellular MTH1, but not many other proteins (Fig. 3a), indicating that NPD7155 and NPD9948 specifically bind to MTH1 in HeLa cells.

Since recent publications suggest that MTH1 is essential for cancer cell survival^8^,^9^, we analyzed the cytotoxic effects of our MTH1 inhibitors against cancer cells. Contrary to our expectations, NPD7155 and NPD9948 exhibited only weak cytotoxicity against HeLa cells (IC_50_ = 65 μM and 35 μM, respectively; Fig. 3b) despite potent MTH1-inhibitory activity with sub-micromolar ranges of IC_50_ values *in vitro* (Supplementary Table S1). In addition, our purine-based MTH1 inhibitors exhibited only weak cytotoxicity in other cancer cell lines (Supplementary Fig. S2). In contrast, (*S*)-crizotinib and SCH51344 had more potent cytotoxic effects (IC_50_ = 13 μM and 5.5 μM, respectively; Fig. 3b), even though these compounds displayed the same degree of MTH1-inhibitory activities as NPD7155 and NPD9948 *in vitro* (Supplementary Table S1).

It has been reported that MTH1 plays a crucial role in preventing incorporation of oxidized nucleotides into nuclear and mitochondrial DNA, allaying subsequent DNA damage^35^. Therefore, we examined the effects of our MTH1 inhibitors on 8-oxo-2′-deoxyguanosine (8-oxo-dG) levels in DNA. However, NPD7155 and NPD9948 did not induce the significant accumulation of 8-oxo-dG in HeLa cells, even at cytotoxic concentrations, when compared to that of TH287 (Fig. 3b and Supplementary Fig. S3a). Next, we investigated whether our MTH1 inhibitors can induce DNA damage, and found that NPD7155 and NPD9948 increased nuclear 53BP1 foci formation, a specific marker for DNA damage, only at the higher concentrations (Supplementary Fig. S3b). Cell cycle analysis showed that NPD7155 and NPD9948 increased the sub-G1 cell population, an indication of dead cells, at the same higher concentrations (Supplementary Fig. S3c). However, NPD8880 also induced DNA damage (Supplementary Fig. S3b) and cell growth inhibition (IC_50_ = 650 μM; Fig. 3b) at similar concentrations in spite of the little MTH1-inhibitory activity (Fig. 1c). These data raised the question whether MTH1 inhibition is responsible for the cytotoxic effects of small-molecule MTH1 inhibitors.

### Mechanistic differences among MTH1 inhibitors

**Figure 4:**
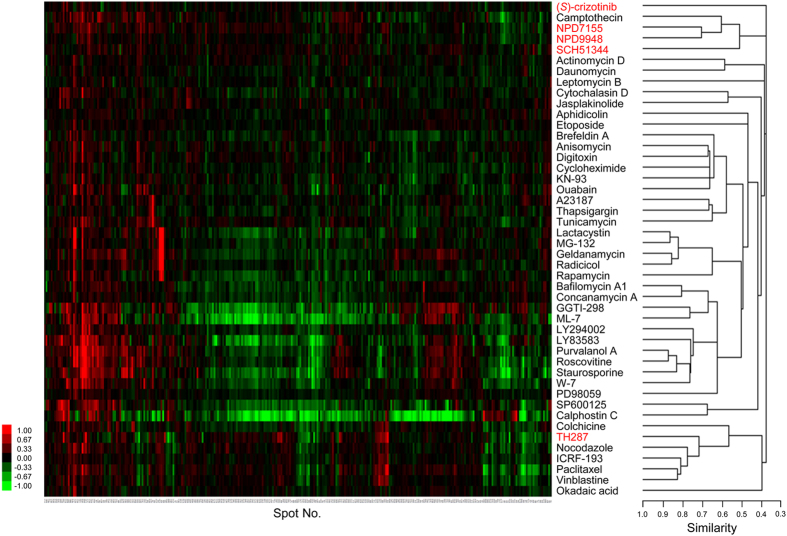
Proteomic profiling shows mechanistic differences among MTH1 inhibitors. HeLa cells were treated with NPD7155 (300 μM), NPD9948 (300 μM), SCH51344 (60 μM), (*S*)-crizotinib (20 μM), or TH287 (3 μM) for 18 h. Proteomic analysis of cell lysates was performed by the 2-D DIGE system. Quantitative data of the common 296 spots (x-axis) derived from MTH1 inhibitors and those of 41 well-characterized compounds were analyzed by hierarchical clustering. As shown in the scale bar for the heat map, intensities of red and green coloration indicate an increased or decreased log-fold (natural base) normalized volume, respectively, of spots for each compound. The vertical axis represents compounds. The horizontal axes of the heat map and dendrogram represent spot number and cosine similarity between clusters, respectively.

In order to validate whether chemically distinct MTH1 inhibitors have the same modes of action for cytotoxicity, we performed a profiling analysis using ChemProteoBase, a comprehensive database of cellular proteomic variations induced by treatment with well-characterized bioactive compounds^31^. The hierarchical cluster analysis of five MTH1 inhibitors and 41 standard compounds revealed that the proteomic variation induced by NPD7155 is similar to that induced by NPD9948 (Fig. 4). Furthermore, these two purine-based MTH1 inhibitors shared similarity with camptothecin (a topoisomerase I inhibitor) and SCH51344. Both camptothecin and SCH51344, as well as NPD7155 and NPD9948, are known to induce DNA damage^9^,^36^. On the other hand, the MTH1 inhibitor (*S*)-crizotinib did not belong to any clusters of the standard compounds in our proteomic profiling system (Fig. 4). More surprisingly, proteomic profiling revealed that TH287, the most potent MTH1 inhibitor we tested (Supplementary Table S1), shared similarity with tubulin-targeting agents such as nocodazole, vinblastine, and paclitaxel, rather than other MTH1 inhibitors (Fig. 4).

### TH287 and TH588 are tubulin polymerization inhibitors

**Figure 5:**
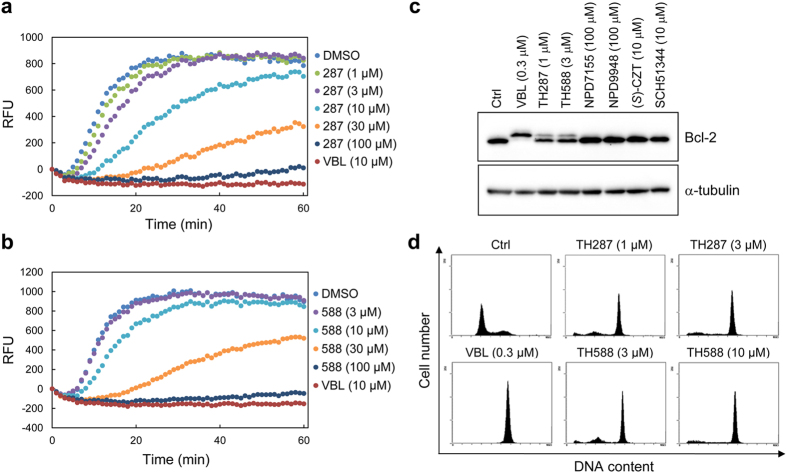
TH287 and TH588 inhibit tubulin polymerization. (**a,b**) Inhibitory effects of TH287 (**a**) and TH588 (**b**) on tubulin polymerization *in vitro*. Vinblastine (VBL) was used as a positive control. (**c**) Detection of phosphorylated Bcl-2 in HeLa cells treated with compounds for 24 h. (**d**) Cell cycle analysis of HeLa cells treated with compounds for 24 h.

Indeed, TH287 and its metabolically more stable analog TH588^8^ inhibited tubulin polymerization *in vitro* in a dose-dependent manner, whereas NPD7155 and NPD9948 did not affect tubulin polymerization, even at 300 μM (Fig. 5a,b and Supplementary Fig. S4). Furthermore, TH287 and TH588 disrupted intracellular microtubule networks in HeLa cells at cytotoxic concentrations, while also inducing cell shrinkage (Fig. 3b and Supplementary Fig. S5). Since tubulin-targeting agents are known to induce phosphorylation of Bcl-2^37^,^38^, we examined the effects of MTH1 inhibitors on Bcl-2. Our results indicated that TH287 and TH588, but not the other MTH1 inhibitors, induced Bcl-2 phosphorylation at the effective concentrations, as detected by the mobility shift on the upper side of unphosphorylated Bcl-2 (Fig. 5c). Likewise, cell cycle analysis revealed that TH287 and TH588, but not the other MTH1 inhibitors, increased the population of HeLa cells in G2/M phase in the same manner as vinblastine (Fig. 5d, Supplementary Figs S3c and S6). These data strongly suggest that tubulin is the primary target of TH287 and TH588, and that it is responsible for their cytotoxic effects.

### MTH1 inhibition does not affect cancer cell growth

**Figure 6:**
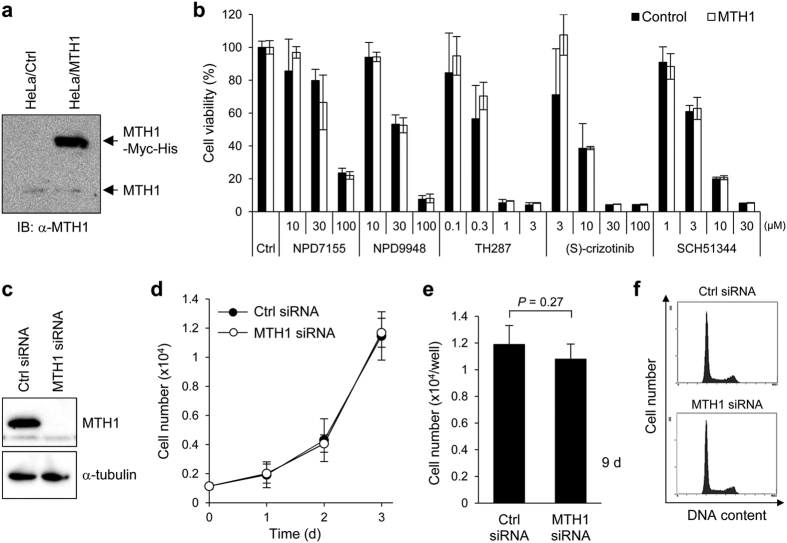
MTH1 inhibition does not affect cancer cell growth. (**a,b**) Overexpression of MTH1 did not rescue cells from MTH1 inhibitors-induced cell death. HeLa cells transiently overexpressing human MTH1 and the control counterparts were treated with MTH1 inhibitors for 84 h. The expression levels of MTH1 proteins in both cells were analyzed by western blot (**a**). The cell viability was examined by WST-8 assay. Data are shown as mean ± s.d. There was no statistically significant difference between the viability of MTH1-overexpressing HeLa cells and that of the control counterparts following treatment with MTH1 inhibitors at all concentrations tested (ANOVA followed by Games-Howell test) (**b**). (**c**–**f**) Knockdown of MTH1 did not affect cell survival. HeLa cells were transfected with 20 nM siRNA for 72 h, and the knockdown of MTH1 was analyzed by western blot (**c**). Cell growth of HeLa cells transfected with 20 nM siRNA for the indicated times (**d**,**e**). Data are shown as mean ± s.d. Statistical analysis was performed using Student’s *t*-test. Cell cycle analysis of HeLa cells transfected with 20 nM siRNA for 96 h (**f**).

As our proteomic profiling showed mechanistic differences among the MTH1 inhibitors, we investigated whether overexpression of human MTH1 rescues cells from MTH1 inhibitor-induced cell death. Contrary to our expectations, MTH1 overexpression did not alter the sensitivity of cells towards any of the MTH1 inhibitors we tested (Fig. 6a,b). Furthermore, siRNA-induced knockdown of MTH1 did not suppress cell growth and cell cycle progression in HeLa cells (Fig. 6c–f). These data suggest that small-molecule MTH1 inhibitors exert cytotoxic effects primarily by targeting off-target molecules such as tubulin, rather than MTH1 inhibition.

## Discussion

In the present study, we found new purine-based MTH1 inhibitors, NPD7155 and NPD9948, that exhibited potent MTH1-inhibitory activity *in vitro* comparable to that of (*S*)-crizotinib and SCH51344 (Supplementary Table S1). However, NPD7155 and NPD9948, showed only weak cytotoxicity compared to that of (*S*)-crizotinib and SCH51344 (Fig. 3b and Supplementary Fig. S2), despite their ability to inhibit the MTH1 activity in intact cells (Fig. 3a). This fact raised the questions whether MTH1 inhibition is responsible for the cytotoxic effects of known small-molecule MTH1 inhibitors and whether MTH1 is suitable as a target molecule for cancer therapy. Indeed, proteomic profiling revealed that TH287, (*S*)-crizotinib, and SCH51344 are mechanistically different (Fig. 4), although these known MTH1 inhibitors showed potent MTH1-inhibitory activity *in vitro* (Supplementary Table S1). In addition, considering that overexpression of human MTH1 did not rescue cells from the cell death induced by small-molecule MTH1 inhibitors (Fig. 6a,b) and knockdown of MTH1 did not suppress cancer cell growth (Fig. 6c–f), the cytotoxic effects of MTH1 inhibitors are probably attributable to their effects on off-target molecules rather than MTH1 inhibition.

Consistent with our observations, two recent reports by Kettle *et al.* and Petrocchi *et al.* independently showed that different classes of MTH1 inhibitors with sub-nanomolar potency inhibited neither proliferation nor survival of human cancer cell line panels^39^,^40^. Kettle *et al.* compared two different siRNA molecules targeting MTH1, one of which, termed “oligo #3,” had been used to demonstrate that genetic ablation of MTH1 leads to reduced tumor growth in a previous study^8^, and found that only oligo #3 suppressed cancer cell survival, although both siRNAs caused significant MTH1 knockdown^39^. Kettle *et al.* also demonstrated that complete knockout of all MTH1 alleles by CRISPR technology did not result in reduced survival of SW480 cells^39^. Together, our data and these reports suggest that tumor growth suppression by genetic or pharmacological inhibition of MTH1 in previous studies was likely due to off-target effects.

The original work showed that TH287 and TH588 did not inhibit other members of the Nudix protein family nor any of the nucleoside triphosphate pyrophosphatases tested^8^. With few exceptions, TH588 had little effect against 87 enzymes, G-protein-coupled receptors (GPCRs), kinases, ion channels, and transporters, even at 10 μM^8^. However, the effects of TH287 and TH588 on tubulin have not been investigated. We know that in general, the concentrations of compounds required for inhibition of tubulin polymerization *in vitro* are higher than those required for disruption of microtubule networks in cells^26^,^30^. In our study, TH287 and TH588 disrupted intracellular microtubule networks and induced Bcl-2 phosphorylation and cell cycle arrest in the M phase at cytotoxic concentrations (Figs 3b and 5c,d and Supplementary Fig. S5), suggesting that tubulin is the primary target responsible for the cytotoxicity of TH287 and TH588 in HeLa cells. In a previous study, TH588 suppressed tumor growth of patient-derived malignant melanoma cells in a mouse xenograft model^8^, but ectopic expression of MutT (a bacterial homolog of MTH1) only partially rescued the cells from TH588-induced cell death^8^. Combined with this report, our findings strongly suggest that TH588 and TH287 exert antitumor effects primarily by inhibiting tubulin.

TH287 increased 8-oxo-dG levels, whereas our MTH1 inhibitors had little effect (Supplementary Fig. S3a). Accumulation of 8-oxo-dG is an indication of oxidative stress, and tubulin-targeting agents such as vinca alkaloids and taxanes are known to induce ROS during the process of cell death^41^. On the other hand, there are complementary enzymes such as 8-oxoguanine DNA glycosylase (OGG1) that minimize the accumulation of 8-oxo-dG in DNA^42^. Although the cause for the different effects of TH287 and our MTH1 inhibitors is unclear, we speculate that these 8-oxo-dG–increasing and –decreasing factors are the determinants of accumulated 8-oxo-dG level.

The mode of action responsible for the cytotoxic effects of (*S*)-crizotinib, SCH51344, and our purine compounds could not be explained through proteomic profiling (Fig. 4). (R)-crizotinib is clinically used as a c-MET/anaplastic lymphoma kinase dual inhibitor^43^, and its (*S*)-enantiomer has also been reported to inhibit c-MET^44^.

Previous publications suggest a key role for MTH1 in *KRAS*-mutated cancer cells^8^,^10^. However, *KRAS*-mutated pancreatic cancer cell lines (PANC-1 and MIA PaCa-2) and *KRAS*-transformed fibroblast cells with elevated ROS levels (NIH3T3/KRAS)^28^ were less sensitive to our MTH1 inhibitors (Supplementary Fig. S2).

In conclusion, tubulin was identified as another target of TH287 and TH588 to cause the cytotoxicity, and MTH1 inhibition alone may not be sufficient for antitumor therapy.

## Methods

### Materials

All compounds used for screening were supplied from a chemical library of the RIKEN NPDepo^32^,^33^. (*S*)-Crizotinib, SCH51344, TH287 hydrochloride, and TH588 hydrochloride were purchased from ApexBio Technology, Santa Cruz Biotechnology, Axon Medchem, and Sigma-Aldrich, respectively. The purity of the compounds was checked using LC-MS.

### Chemical array screening

To prepare the chemical arrays, solutions of the 29,809 compounds (2.5 mg/mL in dimethyl sulfoxide; DMSO) from the RIKEN Natural Products Depository (NPDepo) were arrayed onto 11 separate photoaffinity linker-coated glass slides (using a chemical array-manufacturing apparatus developed by RIKEN) and immobilized via a photo-cross-linking method^14^. The chemical array screening was performed according to our previous reports^17^,^18^. Briefly, we prepared a lysate of HEK293T cells that were transiently overexpressing DsRed-fused MTH1 or DsRed protein in phosphate-buffered saline (PBS), and incubated the chemical arrays with the cell lysate for 1 h at 4 °C. After washing with PBS, the chemical arrays were scanned using a GenePix microarray scanner (Molecular Devices) with an excitation wavelength of 532 nm. The fluorescence signals were quantified using the GenePix Pro 5.0 software (Molecular Devices) with local background subtraction. We used data from slides treated with a lysate of DsRed-expressing cells as a reference. The images from two slides treated with a cell lysate of MTH1-DsRed-overexpressing cells and that of DsRed-overexpressing cells were colored red and green, respectively, using Photoshop 5.5 software (Adobe Photoshop), and merged into a composite image.

### Enzyme assay for MTH1, ITPA, and DCTPP1

An MTH1 enzyme assay was performed as described previously with minor modifications^9^. Briefly, test compounds were dissolved in an assay buffer [100 mM Tris-acetate, 40 mM NaCl, 10 mM Mg(OAc)_2_, 0.005% Tween-20, and 2 mM dithiothreitol (DTT), pH 7.5] containing His-tagged human recombinant MTH1 protein (2 nM; ProSpec). After addition of the mixed reagent from the PPiLight Inorganic Pyrophosphate Assay kit (Lonza Rockland), either 8-oxo-dGTP (13.2 μM; TriLink BioTechnologies) or 2-OH-dATP (8.3 μM; Jena Bioscience) was added as a substrate to initiate the enzymatic reaction. Relative MTH1 activity was measured by monitoring the pyrophosphate (PPi) generated through MTH1-catalyzed nucleotide triphosphate hydrolysis. The luciferase-mediated luminescence signal was recorded for 30 min using a luminometer (Molecular Devices). Then, SigmaPlot (Hulinks) was used for kinetic analysis to determine the inhibition patterns of NPD7155 and NPD9948 and calculate their *K*_i_ values.

Likewise, the enzymatic activities of ITPA and DCTPP1 were measured using a PPiLight Inorganic Pyrophosphate Assay kit. For the ITPA enzyme assay, we used ITPA assay buffer (100 mM Tris-acetate, 50 mM Mg(OAc)_2_, 0.005% Tween-20, and 2 mM DTT, pH 7.5), His-tagged human recombinant ITPA protein (5 nM; ProSpec), and ITP (25 μM). For the DCTPP1 enzyme assay, we used DCTPP1 assay buffer (100 mM Tris-acetate, 100 mM NaCl, 10 mM Mg(OAc)_2_, 0.005% Tween-20, and 2 mM DTT, pH 7.5), His-tagged human recombinant DCTPP1 protein (50 nM; ATGen), and deoxycytidine triphosphate (dCTP; 50 μM).

To exclude false positive compounds that inhibit PPi detection, the effects of test compounds on 10 μM Na_4_P_2_O_7_-induced luciferase activity were examined.

### Target engagement assay

To analyze the interaction between small-molecule ligands and their target proteins in intact cells, a cellular thermal shift assay (CETSA) was performed as described previously with minor modifications^9^,^34^. Briefly, HeLa cells cultured in a 100-mm dish to 80% confluency, were treated with NPD7155 (300 μM), NPD9948 (300 μM), or (*S*)-crizotinib (30 μM) for 1 h. After treatment, cells were collected with trypsin and resuspended in Tris-buffered saline (TBS). The cell suspension was aliquoted into four polymerase chain reaction (PCR) tubes and heated at 45, 50, 55, or 60 °C for 5 min. Subsequently, cells were lysed by three repeated freeze-thaw cycles using liquid nitrogen. Precipitated proteins were separated from the soluble fraction by centrifugation at 17,000 × *g* for 20 min. Soluble proteins collected in the supernatant were subjected to sodium dodecyl sulfate polyacrylamide gel electrophoresis (SDS-PAGE) and transferred to a polyvinylidene difluoride (PVDF) membrane (Millipore) for western blot analysis. The membrane was incubated with a primary antibody against MTH1 (1:500, sc-67291; Santa Cruz Biotechnology) and a horseradish peroxidase–labeled secondary antibody (Vector Laboratories) in sequence. They were then visualized with Fusion Solo S (Vilber Lourmat) using a Super Signal West Pico Chemiluminescence Substrate (Pierce). After detection of MTH1 by western blot, proteins on the membrane were visualized by staining with Coomassie brilliant blue (CBB).

### Profiling by ChemProteoBase

ChemProteoBase profiling analysis was performed as described previously^31^. Briefly, HeLa cells were treated with compounds for 18 h. Proteome analysis of the cell lysate was performed using a 2D difference gel electrophoresis (2-D DIGE) system (GE Healthcare). Images of the gels were analyzed with Progenesis SameSpots (Nonlinear Dynamics). Of the more than 1000 spots detected in each 2D gel, 296 variational spots found to be common between the gels of the reference and compound-treated cells were selected as described^31^. Next, the volume of each spot was normalized using the average of the corresponding control values from DMSO-treated HeLa cells, and the log-fold ratio of the normalized volumes and the average value in triplicate was calculated. The ChemProteoBase dataset was constructed from the data acquired from HeLa cells treated with RIKEN NPDepo library standard compounds. Data for the 296 spots, which were acquired from the test compounds, and the ChemProteoBase dataset were combined, and hierarchical clustering analysis was performed with Cluster 3.0 (clustering method; centroid linkage with the means of uncentered correlation). The predictive dendrogram was visualized with Java Tree View 1.1.3.

### Tubulin polymerization assay

*In vitro* tubulin polymerization assay was performed using a tubulin polymerization assay kit (Cytoskeleton) according to the manufacturer’s instructions. Briefly, lyophilized porcine tubulin was solubilized to a final concentration of 2 mg/mL in reaction buffer containing 80 mM piperazine-N,N′-bis(2-ethanesulfonic acid) (PIPES; pH 6.9), 2 mM MgCl_2_, 0.5 mM ethylene glycol tetraacetic acid (EGTA), 1 mM guanosine-5′-triphosphate (GTP), 10 μM fluorescent reporter, and 20% glycerol, and kept at 4 °C. Compounds (100 × DMSO stock solutions) were added to prewarmed half-area 96-well black plates. Cold tubulin solution was added to the wells. The plate contents were mixed by shaking, and fluorescence (Ex 350 nm, Em 435 nm) was read every minute for 1 h using a fluorescence microplate reader (Molecular Devices).

### Detection of phosphorylated Bcl-2

Western blot analysis for detection of phosphorylated Bcl-2 was performed as described previously with several modifications^37^,^38^. Briefly, HeLa cells were treated with test compounds for 24 h, then harvested and lysed by sonication in a radioimmunoprecipitation assay buffer (RIPA) buffer containing 25 mM 4-(2-hydroxyethyl)-1-piperazineethanesulfonic acid (HEPES; pH 7.8), 0.5 M NaCl, 5 mM ethylenediaminetetraacetic acid (EDTA), 1.5% Triton-X-100, 1.0% sodium deoxycholate, and 0.1% sodium dodecyl sulfate (SDS), supplemented with a protease inhibitor cocktail (Roche). Samples were subjected to SDS-PAGE and transferred to a PVDF membrane. The membrane was incubated with a primary antibody against Bcl-2 (1:500, #M0887; DAKO) and a horseradish peroxidase-labeled secondary antibody (1:2000, 170-6516; Bio-Rad) in sequence. Then, they were visualized with Fusion Solo S using the SuperSignal West Pico Chemiluminescence Substrate. Phosphorylated Bcl-2 was detected on the upper side of unphosphorylated Bcl-2 by the mobility shift. As a loading control, α-tubulin was detected in the same samples by using a primary antibody against α-tubulin (1:2000, T9026; Sigma-Aldrich).

### Overexpression of MTH1

For construction of the pcDNA3.1/MTH1 plasmid, the MTH1 cDNA was cloned from HeLa cells by PCR using the forward primer 5′-AGTGTGGTGGAATTCATGAGTGGAATTAGCCCTCA-3′ and the reverse primer 5′-ATATCTGCAGAATTCGGACCGTGTCCACCTCGCGG-3′, and inserted into pcDNA3.1/*myc*-His vector (Invitrogen) with the *Eco*RI restriction site. The destination plasmid was verified by sequencing analysis. For preparation of MTH1-overexpressing cells, HeLa cells were seeded in 6-well plate, cultured overnight, and transfected with the pcDNA3.1/MTH1 plasmid or the control vector using Effectene Transfection Reagent (Qiagen) according to the manufacturer’s instruction. After 24 h of the transfection, the cells were reseeded in 96-well plate, incubated for 12 h, and then treated with MTH1 inhibitors for 84 h. The cell viability was examined by WST-8 assay (see Supplementary Methods “Cell death assay”). After 24 h of the transfection, the expression level of MTH1 protein was analyzed by western blot (see “Target engagement assay”).

### Knockdown of MTH1

Control siRNA (sc-37007, Santa Cruz Biotechnology) and human MTH1 siRNA (sc-62647, Santa Cruz Biotechnology) were obtained. HeLa cells were transfected with 20 nM siRNA using Lipofectamine RNAiMAX according to the manufacturer’s instructions (Invitrogen). MTH1 knockdown after 72-h siRNA treatment was analyzed by western blot. After transfection, cells were counted using Automated Cell Counter (Bio-Rad).

## Additional Information

**How to cite this article**: Kawamura, T. *et al.* Proteomic profiling of small-molecule inhibitors reveals dispensability of MTH1 for cancer cell survival. *Sci. Rep.* **6**, 26521; doi: 10.1038/srep26521 (2016).

## Supplementary Material

Supplementary Information
