# Supplementary material for: Proteomic profiling of small-molecule inhibitors reveals dispensability of MTH1 for cancer cell survival
Source: Sci Rep. 2016 May 23;6:26521. doi: 10.1038/srep26521 (PMC4876372; doi:10.1038/srep26521)
Supplement: Supplementary Information [file srep26521-s1.pdf]

Supplementary Information for

Proteomic profiling of small-molecule inhibitors reveals dispensability of MTH1 for cancer cell survival

Tatsuro Kawamura[1], Makoto Kawatani[1], Makoto Muroi, Yasumitsu Kondoh, Yushi Futamura, Harumi Aono, Miho Tanaka, Kaori Honda, and Hiroyuki Osada[*]

Chemical Biology Research Group, RIKEN Center for Sustainable Resource Science (CSRS), 2-1 Hirosawa, Wako, Saitama 351-0198, Japan

[1]These authors contributed equally to this work.
[*]To whom correspondence should be addressed: Hiroyuki Osada, Chemical Biology Research Group, RIKEN Center for Sustainable Resource Science (CSRS), 2-1 Hirosawa, Wako, Saitama 351-0198, Japan, Tel: +81-48-467-9541; Fax: +81-48-462-4669; E-mail: hisyo@riken.jp

This PDF file includes:
Supplementary Methods
Supplementary References
Supplementary Table S1
Supplementary Figs. S1 to S7

Supplementary Methods

Cell culture

Human cancer cell lines HeLa (cervical cancer), MIA PaCa-2 (pancreatic cancer), A431 (epidermoid carcinoma), and human embryonic kidney derived cell line HEK293T were cultured in Dulbecco's Modified Eagle's Medium (DMEM; Invitrogen) containing 10% fetal bovine serum (FBS), penicillin G (50 units/mL) and streptomycin (50 μg/mL). Human cancer cell lines Panc-1 (pancreatic cancer) and Jurkat (T cell lymphoma) were cultured in RPMI (Gibco) containing 10% FBS, penicillin G (50 units/mL) and streptomycin (50 μg/mL). Human osteosarcoma cell line MG-63, mouse embryonic fibroblast NIH3T3 cells and its transformant NIH3T3/KRAS cells[1] were cultured in DMEM (Sigma-Aldrich) containing 10% calf serum, glutamine (0.3 g/L), penicillin G (50 units/mL), and streptomycin (50 μg/mL). All cell lines were incubated at 37 °C in a humidified atmosphere containing 5% CO_2.

Cell death assay

The cells were seeded in 96-well plates, cultured overnight, and treated with test compounds for the indicated time. Subsequently, cell viability was measured by the WST-8 assay, using Cell Count Reagent SF (Nacalai Tesque) according to the manufacturer's instructions, with the measurement of absorbance set at 450 nm on the ARVO SX 1420 Multilabel Counter (Wallac).

Immunofluorescence

The HeLa cells were seeded on glass coverslips in 12-well plates, cultured overnight, and treated with test compounds for the indicated time. For 8-oxo-dG, cells were fixed sequentially in 3.7% formalin for 10 min and methanol at -20 ºC for 10 min, and then permeabilized in 0.5% Triton X-100 in PBS at room temperature for 5 min. After blocking with 0.5% BSA in PBS at room temperature for 30 min, cells were incubated sequentially with a primary antibody against 8-oxo-dG (5 μg/mL, MOG-020; Japan Institute for the Control of Aging) and with Alexa Fluor 488-conjugated secondary antibody (1:400, Life Technologies) under the same conditions. After a quick rinse in PBS, DNA was counterstained with 0.1 μg/mL 4′,6-diamidino-2-phenylindole (DAPI) in PBS at room temperature for 10 min. Images were analyzed with a fluorescence microscope (BX60, Olympus).

For 53BP1, test compound–treated HeLa cells were fixed in 3% formalin in PBS containing 0.1% Triton X-100 for 15 min and permeabilized in 0.5% Triton X-100 for 5 min. After blocking with 10% FBS and 0.1% Triton X-100 in PBS for 1 h, cells were incubated sequentially with a primary antibody against 53BP1 (1:600, sc-22760; Santa Cruz Biotechnology) in blocking solution at 37 ºC for 1 h and with Alexa Fluor 568-conjugated secondary antibody (1:400, Life Technologies) under the same condition. After a quick rinse in PBS, DNA was counterstained with 0.5 μg/mL Hoechst 33342 (Sigma-Aldrich) in PBS at room temperature for 10 min. Images were analyzed on a fluorescence microscope (Delta Vision).

For α-tubulin, immunofluorescence was performed as described previously with several modifications[2]. Briefly, test compound–treated HeLa cells were fixed in 3.7% formalin for 1 min and methanol at -20 ºC for 5 min sequentially, and permeabilized in 0.5% Triton X-100 in PBS for 5 min. After blocking with 0.5% BSA in PBS at room temperature for 30 min, cells were incubated sequentially with a primary antibody against α-tubulin (1:200, T9026; Sigma-Aldrich) in the blocking solution at 37 ºC for 1 h, and with Alexa Fluor 488-conjugated secondary antibody (1:400, Life Technologies) at 37 ºC for 40 min. After a quick rinse in PBS, DNA was counterstained with 0.5 μg/mL Hoechst 33342 in PBS at room temperature for 10 min. Images were analyzed using a fluorescence microscope (Delta Vision).

Cell cycle analysis

The HeLa cells were seeded in 12-well plates and treated with test compounds for the indicated time. Cells were harvested, washed with PBS, and incubated in propidium iodide (PI) solution containing 50 μg/mL propidium iodide, 0.1% sodium citrate, 0.2% Nonidet P-40, and 0.25 μg/mL RNase A (Nacalai Tesque) for 30 min in the dark. Cellular DNA content was analyzed by fluorescence-activated cell sorting using a Cytomics FC500 (Beckman Coulter).

Supplementary References

1 Kawamura, T., Kondoh, Y., Muroi, M., Kawatani, M. & Osada, H. A small molecule that induces reactive oxygen species via cellular glutathione depletion. *Biochem. J.* **463**, 53-63 (2014).

2 Futamura, Y. *et al.* Morphobase, an encyclopedic cell morphology database, and

its use for drug target identification. *Chem. Biol.* **19**, 1620-1630 (2012).

Supplementary Table S1. Chemical structures and MTH1 inhibitory activities of purine derivatives and known MTH1 inhibitors.

		IC_{50} of MTH1 inhibitory activity (μM)	
Compound	Chemical structure	8-oxo-dGTP	2-OH-dATP
NPD15095	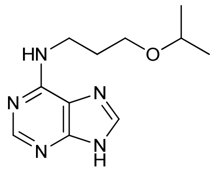	3.3 ± 0.6	6.7 ± 2.0
NPD7155	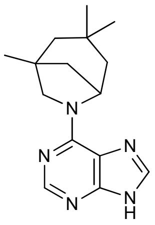	0.21 ± 0.03	0.38 ± 0.16
NPD9948	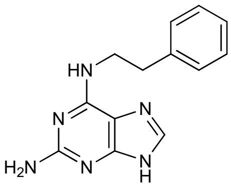	0.29 ± 0.05	0.50 ± 0.21
NPD8880	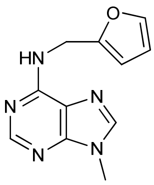	> 1,000	> 1,000
TH287	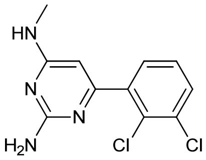	0.0022 ± 0.0003	0.0036 ± 0.0017
TH588	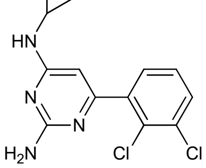	0.011 ± 0.003	0.019 ± 0.008
(*S*)-crizotinib	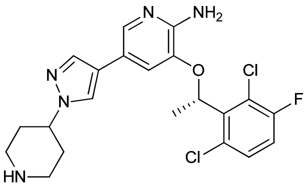	0.39 ± 0.10	0.73 ± 0.33
SCH51344	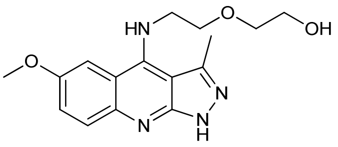	0.32 ± 0.04	0.75 ± 0.40

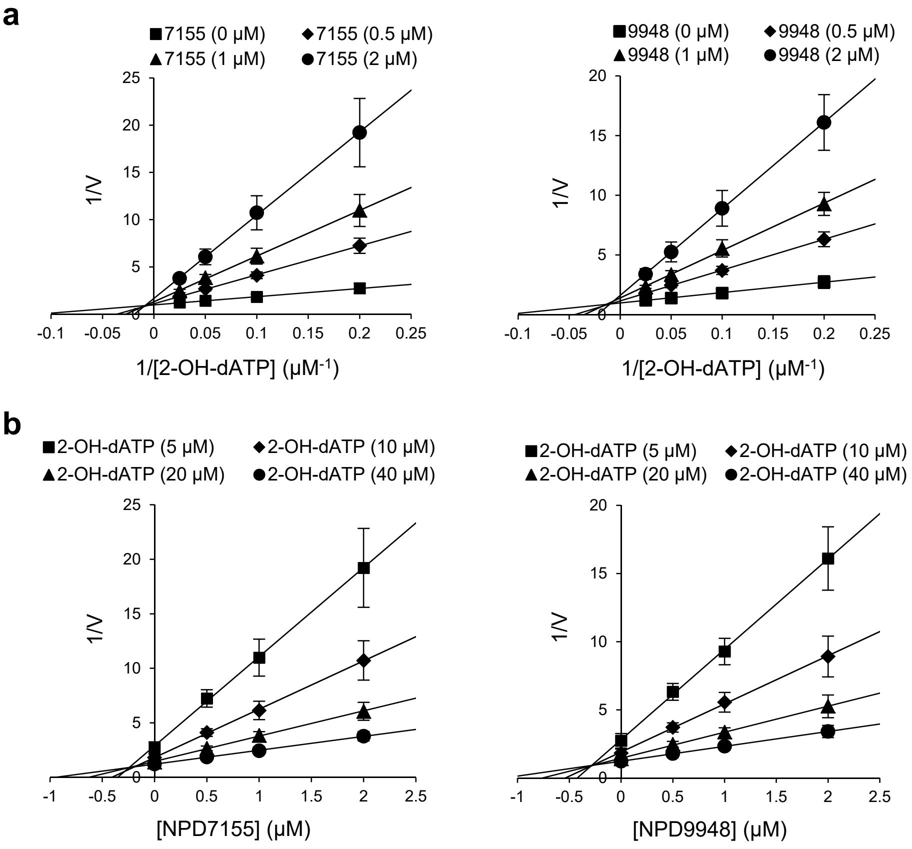

Supplementary Fig. S1. Kinetic analysis for NPD7155 and NPD9948 against MTH1. (**a**) Lineweaver–Burk plot of reciprocal of initial velocity vs. reciprocal of varying concentrations of 2-OH-dATP. 7155, NPD7155; 9948, NPD9948. (**b**) Dixon plot of initial velocity vs. varying concentrations of NPD7155 or NPD9948. Data are shown as mean ± s.e.m. from three independent experiments.

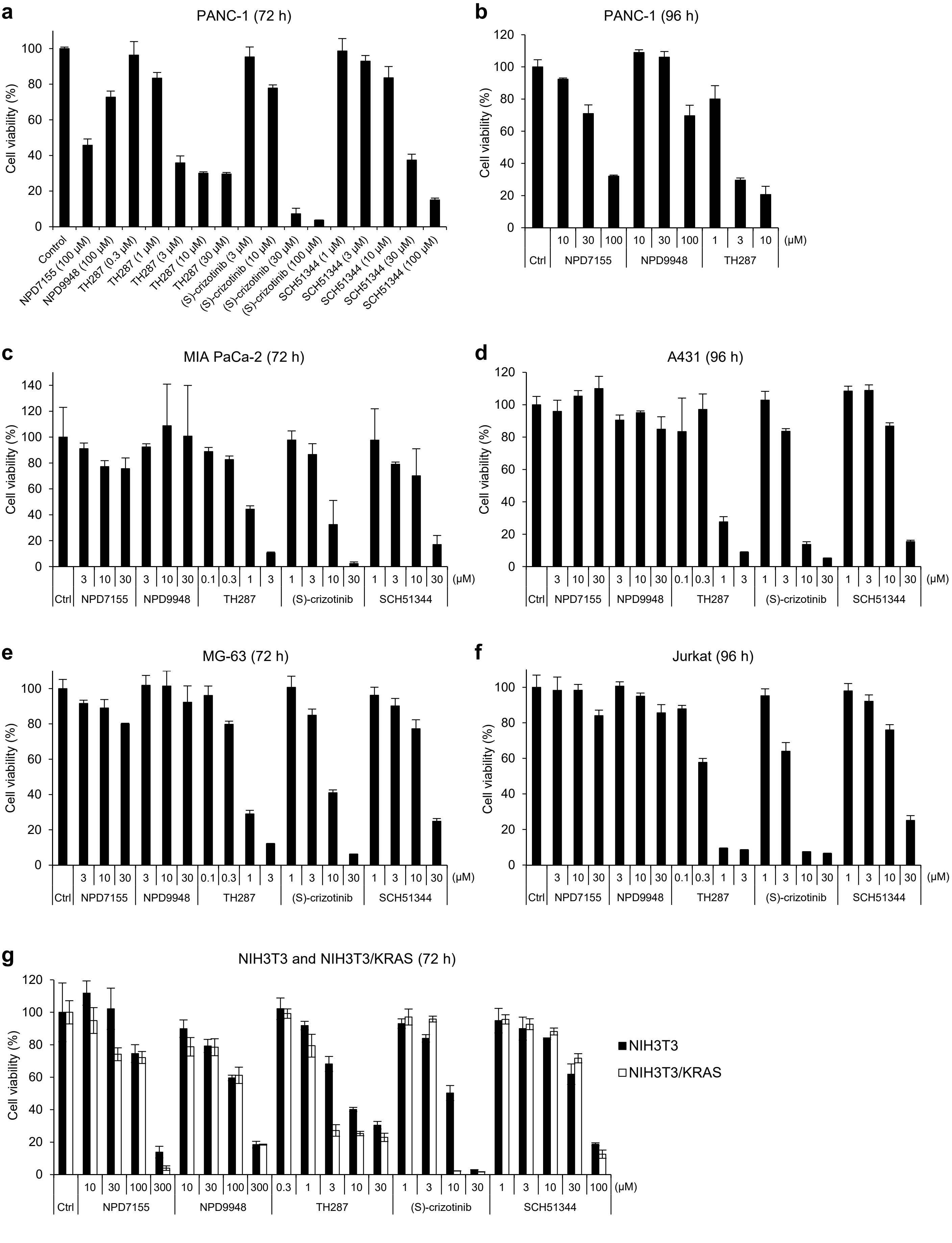

Supplementary Fig. S2. NPD7155 and NPD9948 exhibit less potent cytotoxicity against cancer cell lines and KRAS-transformed fibroblast cells. (**a-g**) The indicated cells were treated with MTH1 inhibitors for the indicated time. The cell viability was examined by WST-8 assay. Data are shown as mean ± s.d.

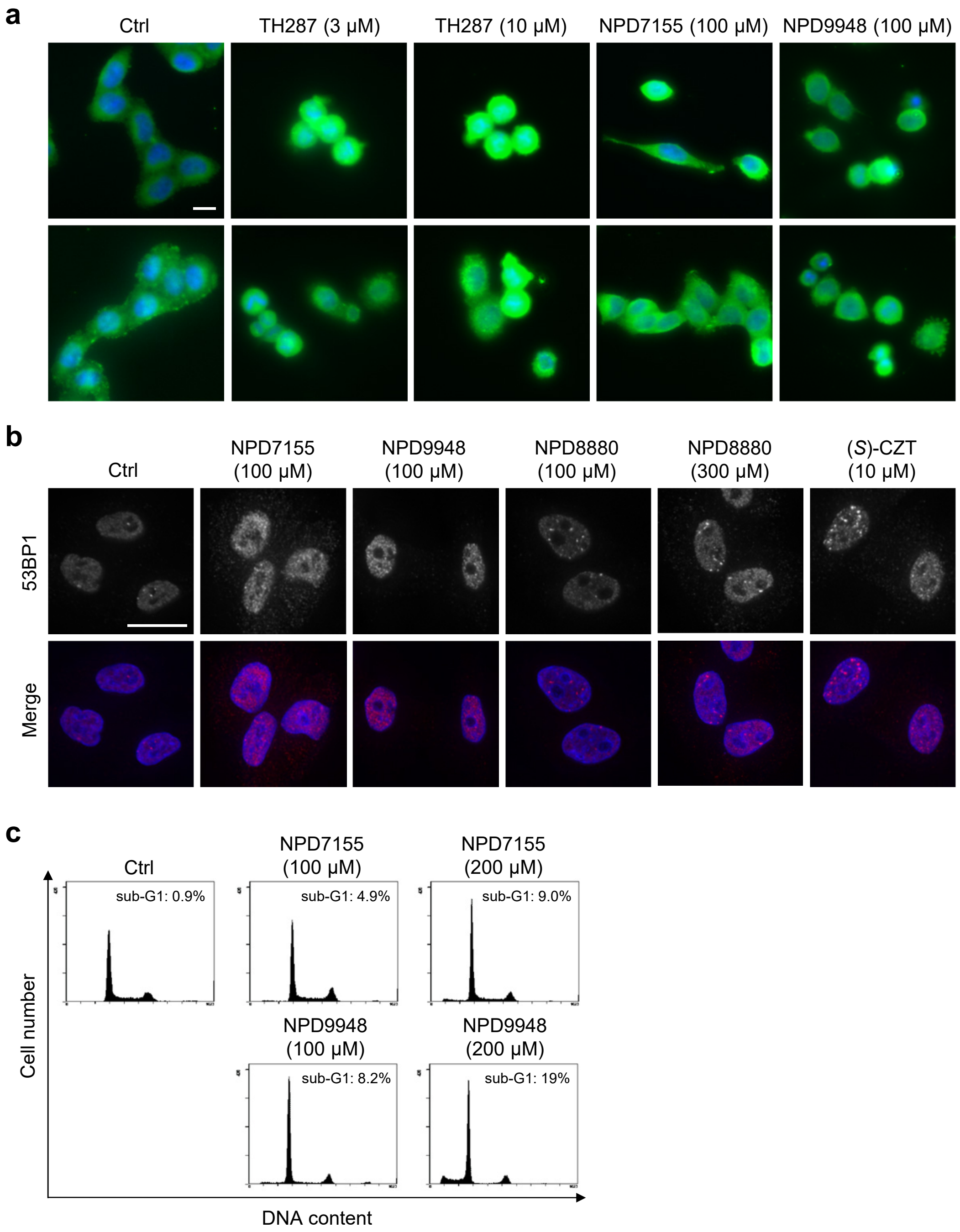

Supplementary Fig. S3. Effects of NPD7155 and NPD9948 on 8-oxo-dG accumulation, DNA damage, and cell cycle progression. (**a**) NPD7155 and NPD9948 hardly induce 8-oxo-dG accumulation. Images of accumulated 8-oxo-2′-deoxyguanosine (green) and DNA (blue) in HeLa cells treated with compounds for 20 h. Scale bar, 15 µm. (**b**) NPD7155, NPD9948 and NPD8880 induce DNA damage. Images of induced 53BP1 foci (red) and DNA (blue) in HeLa cells treated with compounds for 24 h. Scale bar, 15 µm. (**c**) Effects of NPD7155 and NPD9948 on cell cycle progression. HeLa cells were treated with the indicated compounds for 24 h, and analyzed by flow cytometry after propidium iodide staining.

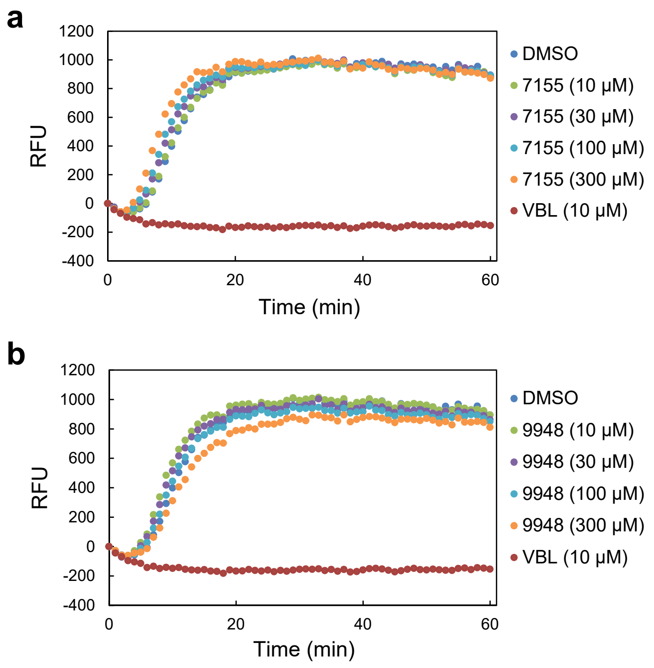

Supplementary Fig. S4. Effects of NPD7155 and NPD9948 on *in vitro* tubulin polymerization. (**a, b**) Effects of NPD7155 (7155) (**a**) and NPD9948 (9948) (**b**) on in vitro tubulin polymerization were determined. Vinblastine (VBL) was used as a positive control.

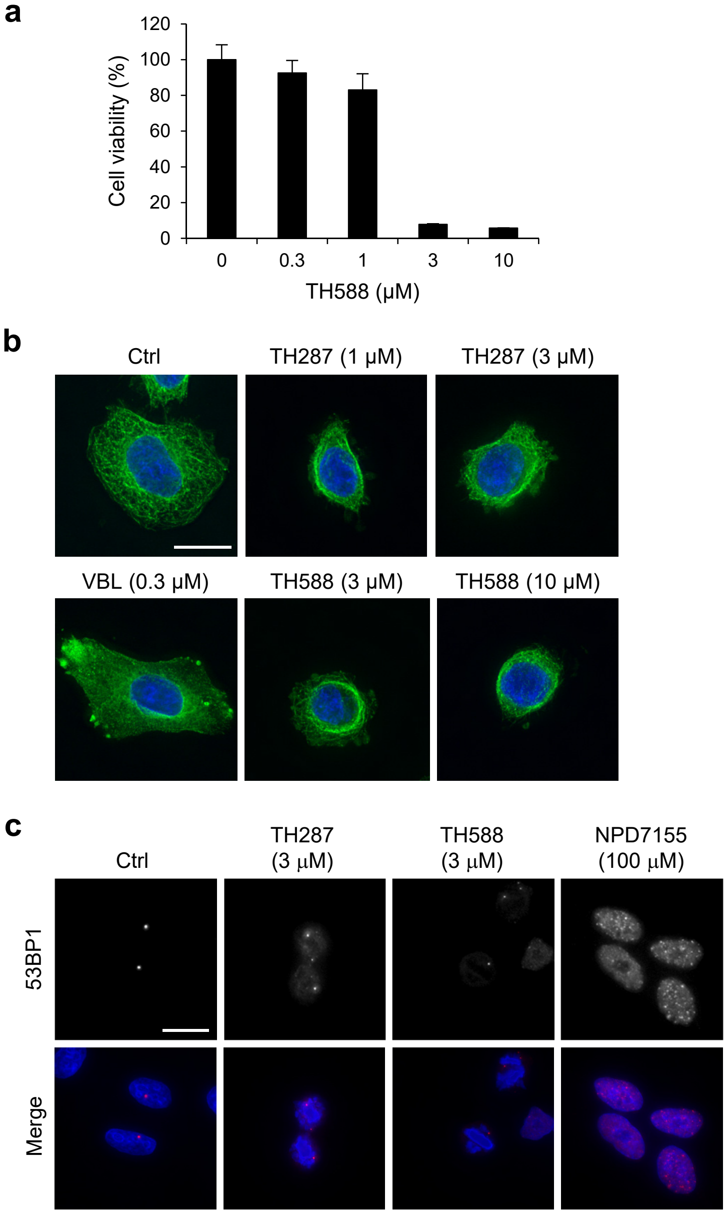

Supplementary Fig. S5. Effects of TH287 and TH588 on interphase microtubules and DNA damage. (**a**) Cytotoxicity of TH588. HeLa cells were treated with TH588 at the indicated concentrations for 72 h. Cell viability was measured by WST-8 assay. Data are shown as mean ± s.d. (**b**) Effects on interphase microtubules. Images of α-tubulin (green) and DNA (blue) in HeLa cells treated with compounds for 6 h. Scale bar, 15 μm. (**c**) Images of induced 53BP1 foci (red) and DNA (blue) in HeLa cells treated with compounds for 24 h. Scale bar, 15 μm.

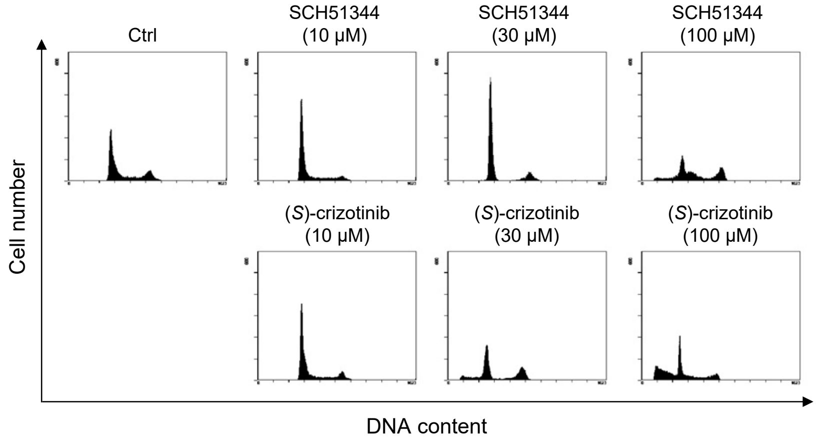

Supplementary Fig. S6. Effects of SCH51344 and (*S*)-crizotinib on cell cycle progression. HeLa cells were treated with the indicated compounds for 24 h, and analyzed by flow cytometry after propidium iodide staining.

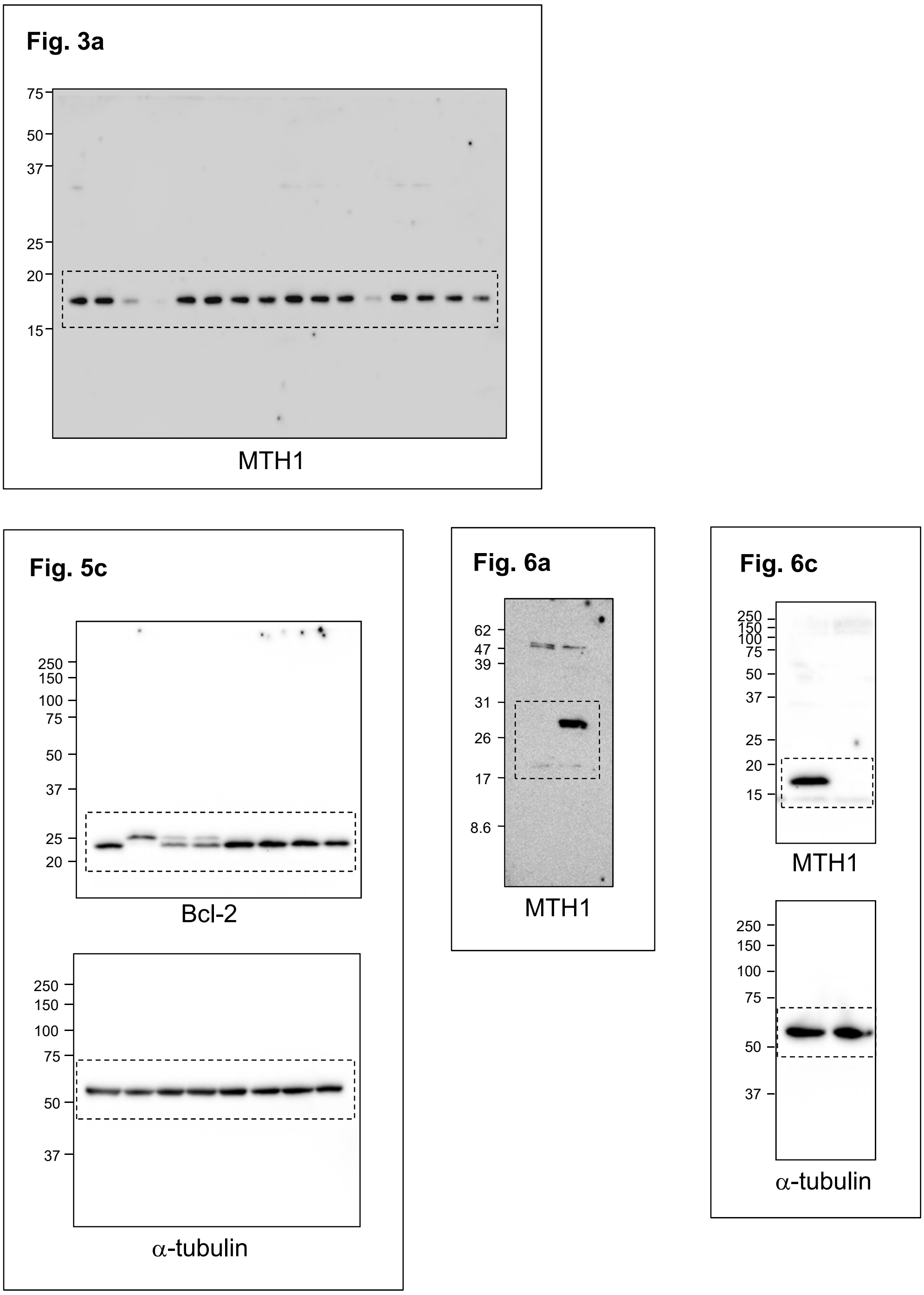

Supplementary Fig. S7. Uncropped images of western blots accompanied by molecular size markers (kDa).
